# Exploring reasoning mechanisms in ward rounds: a critical realist multiple case study

**DOI:** 10.1186/s12913-018-3446-6

**Published:** 2018-08-17

**Authors:** Paul Perversi, John Yearwood, Emilia Bellucci, Andrew Stranieri, Jim Warren, Frada Burstein, Heather Mays, Alan Wolff

**Affiliations:** 1 School of Information Technology, Deakin University, Melbourne Burwood Campus, 221 Burwood Highway, Burwood, VIC 3125 Australia; 2 School of Business, Deakin University, Melbourne Burwood Campus, 221 Burwood Highway, Burwood, VIC 3125 Australia; 3 Centre for Informatics and Applied Optimisation, Federation University, University Drive, Mt Helen, VIC 3350 Australia; 4 Department of Computer Science, The University of Auckland, 38 Princes Street, Auckland, 1010 New Zealand; 5 Caulfield School of Information Technology, Monash University, 900 Dandenong Road, Caulfield East, VIC 3145 Australia; 6 Wimmera Health Care Group, 83 Baillie Street, Horsham, VIC 3400 Australia

**Keywords:** Ward rounds, Medical reasoning, Teamwork, Decision-making, Sense-making, Critical realism, Case study, Causal mechanisms, Program theory

## Abstract

**Background:**

Ward rounds are an important and ubiquitous element of hospital care with a history extending well over a century. Although originally intended as a means of educating medical trainees and junior doctors, over time they have become focused on supporting clinical practice. Surprisingly, given their ubiquity and importance, they are under-researched and inadequately understood. This study aims to contribute knowledge in human reasoning within medical teams, meeting a pressing need for research concerning the reasoning occurring in rounds.

**Methods:**

The research reported here aimed to improve the understanding of ward round reasoning by conducting a critical realist case study exploring the collaborative group reasoning mechanisms in the ward rounds of two hospitals in Victoria, Australia. The data collection involved observing rounds, interviewing medical practitioners and holding focus group meetings.

**Results:**

Nine group reasoning mechanisms concerning sharing, agreeing and recording information in the categories of information accumulation, sense-making and decision-making were identified, together forming a program theory of ward round reasoning. In addition, themes spanning across mechanisms were identified, further explaining ward round reasoning and suggesting avenues for future exploration. Themes included the use of various criteria, tensions involving mechanisms, time factors, medical roles and hierarchies.

**Conclusions:**

This paper contributes to the literature by representing rounds in a manner that strengthens understanding of the form of the group reasoning occurring within, thus supporting theory-based evaluation strategies, redesigned practices and training enhancements.

## Background

Ward rounds are integral to hospital inpatient management across the world. Despite their prevalence reportedly declining [1] they remain a mainstream practice, as evidenced by significant policy documents [2, 3]. Ward rounds are here defined as medical teams travelling sequentially from inpatient to inpatient and stopping at each to discuss, consider and make decisions about the details and overall management of care. Topics commonly addressed during rounds include diagnosis, prognosis and treatment planning.

Despite rounds being central to hospital care for over a century [4], studies of ward rounds are scarce. A literature search in 2014 found only 514 papers compared to 75,264 for the relatively recent topic of laparoscopies [5]. Although research has examined details of rounds, such as communication [6–8], little is known about bedside care-processes or what makes a high quality round [9, 10].

The purposes of rounds centre on practitioner training or the treatment and care of patients [11–14]. Practitioner training, whilst subject to much literature attention [15–17], has been noted to infrequently occur in practice [18]. Studies have either directly shown that care management is the main focus [19, 20] or have implied this through primarily identifying care-related elements as findings [21, 22].

Ward rounds, by definition, involve groups of practitioners discussing, deliberating and decision-making; that is, reasoning together. Literature reviews have examined factors influencing ward round quality [23, 24]. These identified the importance of effective communication, collaboration and standardization of processes. Time constraints present another concern, with benefits accruing from practitioners spending more time with patients [25]. Practitioners’ non-technical skills, such as communication, teamwork and leadership skills, have also been a focus of research [10, 22].

Communication, collaboration, time spent with patients and non-technical skills all highlight the importance of collaborative group reasoning in rounds. Furthermore, practitioners reasoning collaboratively is identified as a central target for reducing medical errors [26]. Hence, collaborative group reasoning was identified as the primary focus of this project.

Conceptualisations of medical reasoning have moved from analytic methods to the dual process theory, which balances heuristic and intuitive type 1 reasoning with analytical and systematic type 2 reasoning [27]. More recently, Naturalistic Decision Making (NDM) theories have been recognised as appropriate [28]. These focus on the cognitive functions of decision-making, sense-making, situation awareness and planning in real contexts such as medical ward rounds.

Viewing reasoning in terms of components, as do NDM models, and considering ward rounds as a type of program suggested the development of a program theory of group reasoning. A program theory is an explicit representation of how the program causes intended or actual outcomes [29] and is typically specified in terms of causal mechanisms [30]. For example, given a patient, some practitioners, a diagnosis and a recommended treatment, the outcome may be to persuade the patient to undertake the treatment, and the mechanism will be how the practitioners go about achieving this. Identifying causal mechanisms of ward round reasoning formed the basis of this investigation.

Mechanism-based explanation in social sciences research has received much attention. Hedström and Ylikoski [31] discussed its history and provided a range of contrasting definitions. For this research, mechanisms are conceived as the activities that actors engage in during the ward round to bring about desired change. Critical realism, discussed in more detail below, is a meta-theory that focuses on discovering causal mechanisms, thus is naturally aligned with the aim of this project.

This research therefore seeks to explain the mechanisms of collaborative group reasoning in ward rounds. Motivated by questions such as ‘how does group reasoning in rounds operate?’ and ‘what enables group reasoning and what detracts from it?’ the purpose of this study is to improve the understanding of ward rounds through exploring the reasoning mechanisms occurring therein.

The next section discusses the critical realist approach which supported a case study methodology employing round observations, practitioner interviews and focus groups. The results section then introduces nine group reasoning mechanisms identified through the data collection and themes associated with the mechanisms. Possible applications of the research, along with limitations and potential future investigations, are then presented.

## Methods

This study explored the role of collaborative group reasoning in ward rounds through a critical realist inspired case study of ward rounds in two hospitals in Victoria, Australia.

### Critical realist theoretical underpinnings

Critical realism (CR), which offers a meta-theory that guides the choice of methodology and methods, was selected as an overarching theoretical framework. CR involves theory-building and provides an appropriate underpinning for case studies [32]. It provides a framework whereby ward rounds, although socially constructed, are assumed to have a logic that objectively exists. This allows for CR to capture benefits and avoid pitfalls of its alternatives, being positivist and constructivist approaches [33]. CR is also increasingly used in health sciences research, such as in mental health and illness [34] and home-dialysis decision-making research [35].

CR assumes a three-layered, nested ontology; the ‘real’, the ‘actual’ and the ‘empirical’ [36]. The real includes entities and their inherent causal structures which exist independent of human thought and which must be inferred from observations by a process of retroduction. Mechanisms, which are causal powers that exist in the real layer, are the focus of attention. These may be activated to cause events, which exist in both the real and the actual layers. Events may then be observed to form experiences, which exist in the empirical layer as well as the real and actual layers [37]. Mechanisms, which are emergent and dynamic [36], are not necessarily activated. Contexts and interactions with other mechanisms are crucial factors in the activation of mechanisms.

### Case study methodology

A case study methodology was adopted, in fitting with CR research [32, 37]. The Acute Care wards of two medium-sized rural hospitals in Victoria, Australia were selected as they provided a context of general medical patients and general physicians, thus avoiding peculiarities associated with specialised wards. Wards were staffed by medical teams consisting of registrars, who are doctors undertaking a prescribed specialist postgraduate training program to seek admission as a Fellow of the Royal Australasian College of Physicians, and interns. Consultant physicians visited the wards every morning and conducted ward rounds with medical teams. Medical students and nurses also attended at times.

Data collection included direct observations of ward rounds. Neither audio nor video recording of rounds was conducted due to potential influences on the round, the expressed wishes of practitioners, and the impracticality of implementation. Handwritten notes were taken concerning details of patient visits, such as the discussion threads and the contributions of different practitioners.

Selected practitioners were invited for interview between rounds in a private meeting room. Interviewees were questioned about details of their practice or about practice generally, with respect to topics associated with the development of the theory at the time of interview. This concords with the iterative nature of data collection and theory development as per the CR approach. Interviews were predominantly unstructured, although sample questions aimed at testing theories were put to interviewees where appropriate. Interviews were audio recorded when agreed (7 out of 17), or handwritten notes taken and subsequently supplemented with additional notes. Focus group meetings involved interactive presentations to medical teams, and the current state of theory development regarding mechanisms informed the content of the presentations. Practitioners were openly invited to comment, expand upon, or criticise the content throughout the presentation. Notes were taken during and after meetings, in addition to one meeting being audio recorded.

Appropriate ethics approvals were sought and obtained. Written consent was obtained from all practitioner research participants. Patients were informed of the presence of the researcher, given an explanatory statement providing them an option of withdrawal and asked for verbal consent. No practitioner or patient declined to participate in the study.

### Integrated analysis/collection process

The research process consisted of three stages. These were not predetermined but continued until saturation was reached, as described below. The data was continuously coded, analysed and recoded during and between stages, thus integrating data collection with analysis as per CR research techniques [32].

Mechanism descriptions were presented to participants, in interviews and focus groups verbally and through diagrams and textual descriptions, who were able to confirm, elaborate on, modify or refute them as appropriate. Presentations and group discussions occurred within the research team, again to substantiate the developing theories. This continued, interspersed with data collection, until a clear picture emerged of the mechanisms occurring in rounds, as determined through broad agreement amongst the participants and the research team.

Validity and generalization are common problems with case studies [38]. These were dealt with through the triangulation of observations, interviews and focus groups, and by grounding the model in well-established theories in domains such as medical reasoning, NDM, group reasoning and ward rounds. Case studies can also suffer from overly complex results [39]. This was addressed through integrating analysis with data collection, whereby practitioners were continually engaged in the development of the mechanisms, thus ensuring that results were clear and understandable.

## Results

### Ward round group reasoning mechanisms

The data collection occurred between August 2015 and January 2017. Eleven rounds were observed, consisting of 94 patient visits involving 7 consultants, 12 registrars and 11 interns. Only one consultant was female, although the gender balance of registrars and interns was approximately equal. Consultants varied in age and experience and in historical service, with 5 of the 7 being trained outside Australia. Patient visits varied from 5 to 20 min and typically involved a practitioner discussion outside the room, a visit to the patient, then a concluding discussion again outside the room. Fifteen practitioners and 2 students were interviewed, and 4 focus groups facilitated. The focus groups occurred during the regular meeting time and consisted of all medical practitioners at the site.

Nine group reasoning mechanisms were identified. Mechanism construction was initially guided by the medical reasoning literature. Studies typically describe gathering information, understanding the case through forming diagnoses and making treatment and care decisions as key activities of reasoning [29], suggesting three broad categories as the starting point.

**Fig. 1:**
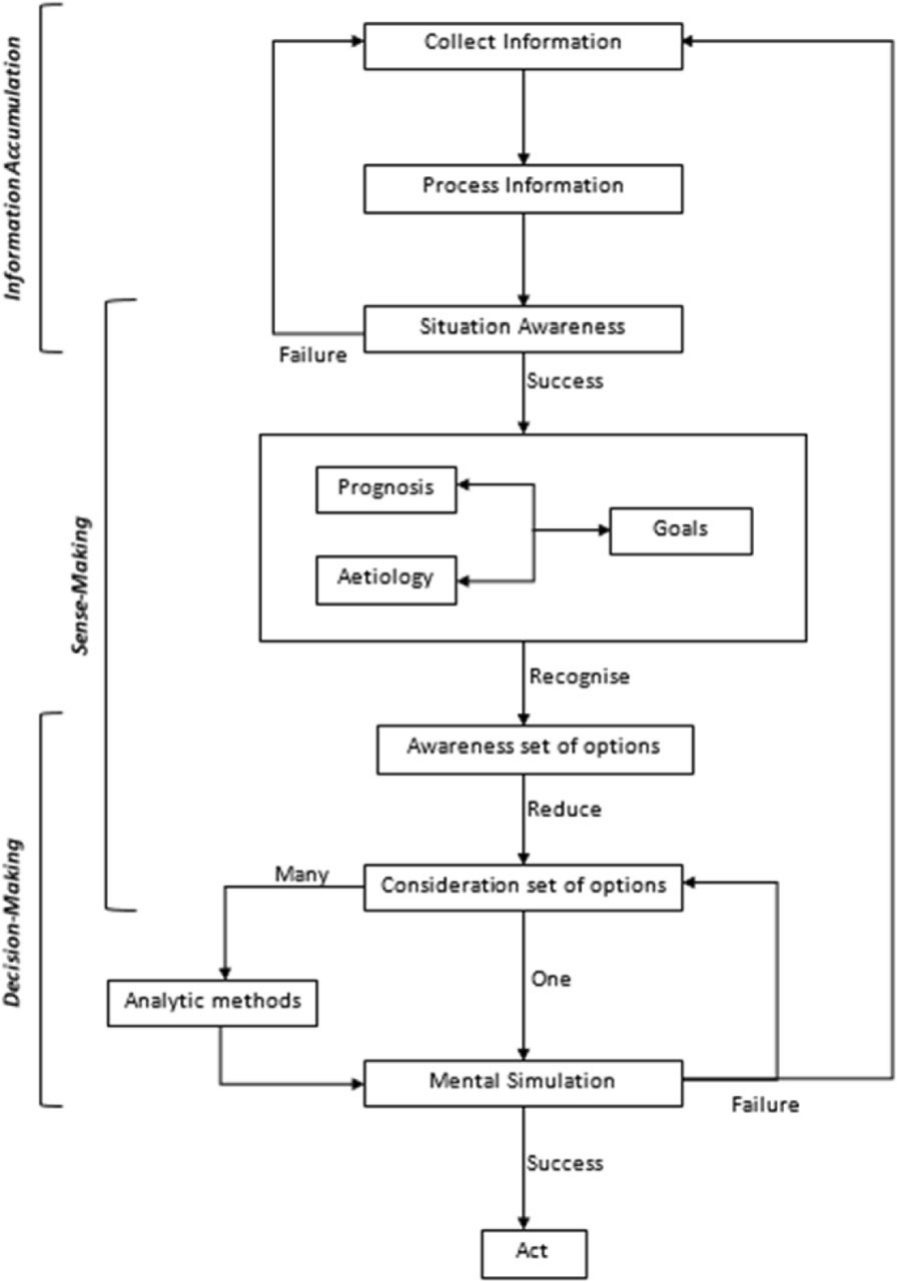
A Naturalistic Decision Making model of ward round reasoning, adapted from Klein [40]

The NDM models mentioned in the introduction, particularly those of Klein [40], provided further confirmation of the three categories: information accumulation, sense-making and decision-making. They also suggested nuanced aspects of reasoning to inform mechanism details, such as mental simulation in decision-making and expertise-based recognition in sense-making. Figure 1 adapts Klein’s synthesized model and overlays it with the three abovementioned mechanism categories.

Mechanisms were formed around the group dimension of ward round reasoning, as the group dimension is what distinguishes ward round reasoning from individual practitioner reasoning. As has been noted, understanding team processes is essential to the functioning of practitioners in collaborative environments [41].

Further considerations involved the connections between reasoning, medical knowledge and roles. Ward rounds are a structured activity and practitioners hold specific knowledge through their roles. Domain knowledge commonly arises in medical reasoning research [42, 43] and the importance of this dimension is further highlighted by models of group reasoning such as the Generic/Actual Argument Model of Yearwood and Stranieri [44].

Incorporating the above considerations, three distinct mechanisms in each of the categories were identified. Table 1 presents the nine mechanisms, all of which convert individually-held information to group-held information.

**Table 1:** The nine collaborative group reasoning mechanisms

	Information Accumulation	Sense-Making	Decision-Making
Sharing	A: Sharing Information	D: Sharing Understanding	G: Sharing Decisions
Agreeing	B: Agreeing About Information	E: Agreeing About Understanding	H: Agreeing About Decisions
Recording	C: Recording Information	F: Recording Understanding	I: Recording Decisions

The remainder of this section outlines each of these mechanisms in turn, as indicated in the body of the table. Each is described, followed by evidence supporting the description. CR asserts that mechanisms exist as causal structures but at times are not activated. Many examples arose of how mechanisms may fail and some are described. The exposition is selective and illustrative, as a complete detailing is beyond the scope of this paper.

### A: Sharing information

#### Description

Participants share individually-held information, verbally or visually through writing, diagrams, images, gestures or exposing signs. Shared information is associated with roles and the central subject is the patient. Participants contribute information either voluntarily, in response to guided questions or through a generative process. Information perceived as relevant and important is contributed, counterbalanced by factors such as sensitivities. Sought information is automatically relevant.

#### Evidence

The data collection identified the patient as central to information collection. Practitioners rarely consulted external information, except for one intern who used his phone to access the internet (patient 5 visit).

Roles influence what information is shared. *‘… interns and registrars often know more on the floor stuff about the patient … then the consultants generate more understanding or experience and it kind of all works together’* (interviewee 13, intern).

Relevance and importance are criteria for sharing. ‘*If you decide a piece of information is relevant then you put it to the group, then the group as a whole decides what’s relevant* … *often will just run things past the other members of the team to say, will that be important …?’* (interviewee 6, intern).

#### How the mechanism fails

Failures occur when practitioners judge information to be irrelevant or unimportant, potentially resulting in a delayed or incorrect diagnosis. ‘*There are situations where you are not sure … you may think it is irrelevant when it is, or relevant when it is not … If you don’t think it is important when it actually is, that’s when things get bad’* (interviewee 8, intern).

### B: Agreeing about information

#### Description

The group agrees about admitting information if practitioners together determine that it passes certain criteria thresholds, such as relevance, importance and reliability. Agreement may be explicit or tacit. Senders and receivers both influence agreement. Objective information is likely to be agreed. High-authority practitioners influence agreement. Information changes agreement status as further shared or individual information arises.

#### Evidence

Agreed information must first be shared. Agreement criteria include source-related factors, ‘… *you have different doctors, different consultants, ... If the registrar is very good … then I will trust them and believe what they tell me. But I’m always very sceptical …’* (interviewee 5, consultant).

Information is often distrusted thus needs to be continually tested. *The patient will give one history to the registrar but then change it for the consultant* (notes from interview 9, consultant).

Authority partially determines whether or not to agree with information. *‘… consultants like to … take a brief history on their own, … and I don’t think I would ever go add more things to the history or ask questions that the consultant or registrar hadn’t thought to ask’* (interviewee 11, intern).

#### How the mechanism fails

The provider of the information may not be trusted or respected. Examples were provided of situations where information from other practitioners was not reliable. ‘*If I … believed everything the registrar was saying, I’m responsible for my actions, for that patient it’s not good ... You’ve got to be careful, you’ve got to have an idea about what the registrars are like’* (interviewee 5, consultant).

### C: Recording information

#### Description

The scribe, usually the most junior medical practitioner, documents shared or agreed information flexibly but within broad recording-practice parameters. Forms provide structure, which direct and constrain recording. Discursive notes are guided by medical formats, but the content often reflects the scribe’s idiosyncratic understanding of various inclusion criteria. Scribes also actively clarify what to record and how to record it. Information is omitted on various grounds, such as sensitivity.

#### Evidence

The intern was typically responsible for scribing. Formats influenced information recording. *‘… most people’s notes, even though they are transcribed a little bit differently, they are often set out in terms of issues that a patient has presented with …’* (interviewee 13, intern).

Recording practices vary considerably. ‘*… you are repeatedly taught things like the pertinent information that you should be capturing, but the way that you actually present that on paper is a very subjective thing. Each person develops their own way’* (interviewee 13, intern).

Sensitive information is sometimes not recorded. *‘… if someone has a difficult family member … you’d probably just verbally give a heads up … you would probably write a … reading between the lines, type of thing in the document …’* (interviewee 11, intern).

#### How the mechanism fails

Quality factors of scribing can undermine information recording. *‘… sometimes you can’t actually decipher what someone’s written … and you don’t do something because you couldn’t read it, couldn’t understand it …’* (interviewee 11, intern).

Difficulties in understanding the case, time constraints and experience levels may hamper appropriate recording. *‘…you are working with time constraints and things like that, so you don’t have time to document everything or every thought process … it is also not practical’* (interviewee 14, registrar).

### D: Sharing understanding

#### Description

Participants share their understandings through verbalising opinions and the reasons for holding them. Understandings involve diagnosis, prognosis, aetiology and appropriate plans. Pre-existing understanding is modified by shared or individually obtained information. Contributions may disrupt existing shared understanding. Practitioners must recognise aberrant information, assess the degree of disruption and judge whether or not to modify or reject the existing shared understanding.

#### Evidence

The basis for sharing understanding involves finding common ground. *‘… I’ll … quickly read the notes for the patients that got admitted before the ward round so that everyone’s on the same page, so generally everyone has the same starting point*’ (interviewee 12, intern).

Sharing understanding relates to authority issues and to knowing the other practitioners’ capacities. *‘Where there are multiple registrars there’s a bit more discussion … but it sort of stops with the consultant … it is a lot to do with … getting to know the others, to see if you feel confident with what they’ve decided’* (interviewee 6, intern).

Hierarchies, whilst shaping involvement, do not prevent participants from contributing. ‘… *often our interns contribute … they come up with suggestions of things we should look for or investigate’* (interviewee 14, registrar), ‘*I encourage a discussion ... You’ve got to be a bit of a dill if you think you’re the only guy who knows anything*’ (interviewee 5, consultant).

#### How the mechanism fails

Junior members lack confidence or believe they have little more to offer, which may cause them to refrain from contributing. ‘*… as an intern we are probably a bit more hesitant, particularly on ward rounds, to contribute, particularly than the registrar would be, the consultant would probably look to the registrar first’* (interviewee 6, intern). *‘Not much gets past the registrars’* (interviewee 11, intern).

### E: Agreeing about understanding

#### Description

Practitioners reach agreement through discussions against a background of shared knowledge. They evaluate the shared understanding using criteria such as whether or not the patient is improving. Agreement is often based on judgement rather than technical argumentation, although understanding reasons is important. Authority is sometimes exercised to influence agreement, often through the senior practitioner’s experience base for pattern recognition. The group’s agreement may be tacit and pseudo-agreement may arise where practitioners defer to others.

#### Evidence

Agreeing about understanding involves information consistency and whether the patient is improving or not. ‘… *when the history and the exam and the investigation findings are all matching up ... when the patient is improving then you can go, yep, we’re on the right track’* (interviewee 6, intern).

Authority is a factor in agreement. When asked whether there are situations where nobody can agree on the problem: ‘*… that does happen, and depending on who it is, it will be whether it is discussed more … often it will be the consultant having a more … dominant role’* (interviewee 6, intern).

Judgement is a significant factor in agreeing, and judgement is associated with authority. *‘There needs to be someone with knowledge and experience … and to make a diagnosis is not as simple as going to the books ... It’s not like mathematics, where 3 plus 3 equals 6’* (interviewee 5, consultant).

#### How the mechanism fails

Individual factors related to experience and personality may interfere with agreement. *Medicine is very complex and expertise varies a lot. The consultant might think something is right but the registrar may disagree. It depends on individual factors too, as experience does not necessarily make one better at making judgements. Other factors involve how outgoing or assertive the registrar is, and the same for the consultant* (notes from interview 16, registrar).

### F: Recording understanding

#### Description

The scribe records the group understanding, usually towards the end of the round, including reasons for that understanding where appropriate. The recorded understanding has been shared but may not be explicitly agreed. Diagnosis, aetiology and prognosis are key topics. Excluded diagnoses with reasons are often recorded, particularly if other recorded information is contradicted by the exclusion. The criteria and structure for recording are applied idiosyncratically, although within general standards.

#### Evidence

The scribe records the group’s understanding but this varies according to the scribe’s features. *‘… the consultant may have an impression about one of these presenting issues and I will always document that ... it’s just a personal thing, it’s who’s scribing at the time’* (interviewee 13, intern).

The information recorded is that which has been agreed by the participants. *‘… there is an agreed shared understanding often and that’s what’s documented …’* (interviewee 12, intern). At the same time, the agreed story is influenced by what has been recorded *‘… what is documented, which ultimately becomes our sort of gospel, is the collaborative story*’ (interviewee 11, intern).

Reasons behind the understanding may be recorded. ‘*… the diagnosis that you decide is not going to be that important than that everyone decides why you came to that*’ (interviewee 6, intern). This may be in a negative form, such as excluded diagnoses. *‘So everybody else knows that you have asked those questions to rule out certain pathologies that need to be ruled out, even if it doesn’t seem immediately pertinent to their presentation’* (interviewee 13, intern).

#### How the mechanism fails

The practitioner authorising the decision may not verbalise enough reasoning to support the scribe’s understanding, and the round moves too quickly to allow for clarification or explication. *‘I remember when I was a junior sometimes registrars would rush through patients and I’d want to ask them more questions or would want to go back… often you’d feel frustrated’* (interviewee 14, registrar).

### G: Sharing decisions

#### Description

Participants share proposed options, their reasons for holding them and associated opinions. Options arise though shared understandings. Biomedical options rely on technical knowledge and experience. Non-medical options are contributed more democratically. Senior practitioners contribute voluntarily or through responding to questions, whereas junior practitioners contribute more discreetly by asking questions in the guise of education or clarifying notes. Practitioners initially share options, which are then shared with patients. If patients find these unacceptable, more options are generated and shared.

#### Evidence

Option contribution takes different forms, depending on who is contributing. *‘… sometimes you can do a sneaky prompt ... if you look in the medication chart and say, oh do we still want the whatever drug, and then they’ll so, oh no, stop that’* (interviewee 12, intern).

Decision type influences options sharing, with less medical topics allowing more democratic contribution. *The case was ‘social’ ... The consultant … asked team members what they thought. The head of rehabilitation … contributed. An OT arrived and also contributed. The nurse manager arrived and contributed re her discussions with the patient’s daughter* (notes from patient 93 visit).

A two stage process of decision-making influences option sharing. ‘*it’s almost two different decisions. You make a decision as a team, and then you take that decision to the patient and you give the options from what the original decision is, as to whether the patient is on board’* (focus group 4, registrar).

#### How the mechanism fails

The atmosphere may not be conducive to practitioners contributing options openly. *‘… each doctor has their own opinions … they don’t think that that is something that is relevant or will work … they’re on a one track mind … if you’ve worked with them for a while and you know what they’re going to think … sometimes people think, it’s not worth arguing about it’* (interviewee 6, intern).

### H: Agreeing about decisions

#### Description

The group agrees on the most suitable option and the reasons for this choice through discussing likely effects. Compensatory and serial decision-making methods are used, both employing mental simulation. Subordinate practitioners defer to the lead practitioner’s authority and agreement may be tacit. Medical and non-medical dimensions allow for varied input by different practitioner roles. Patient agreement occurs after practitioner agreement in a two-stage process, where practitioners present selected options to patients, who ultimately have the right of veto.

#### Evidence

Agreement at times is passive. *‘Sometimes … all we do is agree with it’* (interviewee 5, consultant) and involves mental simulation of the future. *‘… someone … might say, they’re on this medication or they’ve got a past history of this and if we do that then this will happen …’* (interviewee 6, intern).

Practitioners agree between themselves and present a united front to the patient. ‘*… we can’t have a conflict when we go to the patient, right. We should agree to one particular thing and then we present that to the patient*’ (focus group 4, consultant).

Junior practitioners have a significant role in contributing to option evaluation. ‘*Most of the time we will be understanding where the consultant is heading … and hopefully most of the time we will know if there’s anything that is relevant that they’ve missed, like they’ve had a reaction to that, or they’ve got these other comorbidities that will influence them*’ (interviewee 6, intern).

#### How the mechanism fails

Agreement may fail because practitioners have discordant outlooks and cultures. *Surgeons and physicians often have trouble agreeing. They have different paradigms and it depends on the consultants’ personalities, their backgrounds and their mood on the day. It is the least reliable part of the round.* (notes from interview 10, consultant).

### I: Recording decisions

#### Description

The scribe records decisions requiring action by the medical team, other practitioners or the patient. Standard medical practice provides guidance on what is recorded and how it is recorded. Proformas also influence how and what is recorded. Understanding the decision is critical to scribing. Reasons for decisions made are recorded where appropriate, erring on the side of recording if in doubt. Information about excluded treatment options is also recorded.

#### Evidence

Reasons for a decision should be recorded. *‘… if it was a treatment that was obvious, and there was a reason why you weren’t doing … the usual treatment, then most people would document why … because … people will look for that information …’* (interviewee 12, intern).

Content may be consistent even if the format varies. *‘… the way that you actually present that … is a very subjective thing … I write notes completely differently to X and to Y. All same information is probably there …, but it is presented in a very different way …’* (interviewee 13, intern).

#### How the mechanism fails

Multiple factors influence failure to record appropriate information. *‘… they are not comfortable asking what should I write here … it’s not like purposeful, or it’s not ignorance, it’s not the inattention, not the lack of knowledge, it’s multiple things, it’s not simple really’* (focus group 4, consultant). Practitioners may not be able to explain the decision adequately. *‘… sometimes that conversation is quite complex and it doesn’t really translate on the page, and that’s always the difficulty of the complexity of medicine’* (focus group 4, registrar).

### Themes associated with mechanisms

Themes concerning the mechanisms also arose, involving time constraints, hierarchical roles of participants, the use of criteria and tensions concerning mechanisms.

#### Time factors

Time constraints influence how thorough the recording is, how much detail the scribe picks up, when to stop collecting information and when to cease the patient visit. But time is also part of the overall rationale for ward rounds, as rounds allow for efficient task allocation and coordination. *‘… the aspect of doing things, the mechanics of organising and talking to people, organising tests, and that actually takes a fair amount of time … junior staff usually do that’* (interviewee 5, consultant).

#### Hierarchy and roles

Hierarchical roles are associated with tasks, such as interns scribing and consultants overseeing decisions. As noted by a consultant, *‘some doctors won’t make that decision because they’re afraid they’ll be wrong … someone has to make a decision ... That’s why they get characters like me … to make these decisions’* (interviewee 5, consultant).

#### The use of criteria

Relevance is a key criteria for information accumulation but it also arises in other mechanisms, *‘some problems they come in with are more relevant than others’* (interviewee 5, consultant). Relevance also has a contextual dimension, *‘… going through the process of saying why or why not it’s relevant to a particular patient’* (interviewee 6, intern). Many other criteria arise, such as importance, ‘*we’re not 100 percent sure what is important or what is most relevant’* (interviewee 6, intern). Other criteria include consistency, accuracy, truth-value and making sense.

#### Tensions concerning mechanisms

The mechanisms suggest many areas where a balance needs to be reached for effective reasoning.

#### Information collection versus generation

Information is gained through distinct processes. Patients provided specific, sought information, such as through physical examinations or questioning about medication regimes. Alternatively, interactive generation occurred, whereby questions and responses were interpreted idiosyncratically which generated further questions and responses, and so on. This occurred with one patient regarding the circumstances around an unconscious collapse (patient 36).

#### Information accumulation versus sense-making

At some point, information accumulation must stop and making sense of the case can prompt cessation*. ‘All the information has come together so you think, ok, we can stop looking for other things.*’ (interviewee 6, intern). The process of making sense of the case relies on information, but excessive accumulation distracts from sense-making and swamps practitioners with information.

#### Constructing understanding versus disrupting understanding

Two sense-making sub-mechanisms identified were constructing and disrupting understanding. Potential disruption through exposure to critical scrutiny is a critical dimension of sense-making but excessively searching for disruptions will undermine the construction process. ‘… *the information, you can’t have too much but you have to be sensible’* (interviewee 5, consultant).

#### Making sense versus making decisions

Balancing understanding the case and choosing a course of action is also important. *‘Ultimately you want … a diagnosis, and treat with whatever the treatment for that diagnosis is. So that’s your goal. Otherwise, you try and fix what you can’* (interviewee 8, intern). At some point, sense-making must cease and decisions be sought, regardless of how fully the case is understood.

#### Creating options versus evaluating options

A balance also occurs between raising options and evaluating options. Interviewees indicated that numerous options are concurrently evaluated. *‘There’s a myriad of options ... You simultaneously weigh up all of those. I don’t think you go through one, yes or no, two, yes or no’* (interviewee 13, intern). Practitioners cannot fully investigate every possible option, nor continually raise further options, and serial option evaluation is sometimes required.

#### Group versus individual

Sole practitioners can ameliorate biases, knowledge gaps and reasoning limitation through group reasoning. But practitioners are also individually liable, thus must balance group reasoning benefits against risks. ‘*I can tell you people make mistakes, when they feel that all that they’re told is the truth … before I do anything I always have a look at it myself.*’ (interviewee 5, consultant).

#### Nomothetic versus idiographic knowledge

Both sense-making and decision-making require balancing nomothetic and idiographic knowledge. Consultants bring nomothetic knowledge to the group, *‘… perhaps it wouldn’t be sensible to … do any sort of exotic surgery, but those decisions … are very difficult and usually the senior physician will do that’* (interviewee 5, consultant). Interns contribute idiographic knowledge, *‘… you know your patients inside out, because you see them every day, you were there when they were admitted and you have a much better understanding of the clinical picture’* (interviewee 13, intern).

## Discussion

### Significance

This study has conceptualised the collaborative group reasoning that occurs in ward rounds in terms of causal mechanisms. The critical realist inspired approach has resulted in the identification of 9 group reasoning mechanisms and several themes related to those mechanisms. To our knowledge, this is the first time that any attempt to characterise ward round reasoning in such a way has been attempted.

As mentioned above, better non-technical skills, such as communication and teamwork, have been called for in rounds [10, 22]. The mechanisms help to identify how and where such skills may be utilised to the greatest advantage. For example, practitioners sharing their understandings require strong communicative skills, whereas agreeing on understanding requires strong cooperative teamwork and negotiation skills. These skills can be viewed in relation to tasks associated with hierarchies and roles. These may be mapped for each mechanism, and the interactions between mechanisms with respect to roles and tasks explored. An example is the connection between note-taking and sense-making, where senior practitioners can partially dictate the team’s agreed understanding of the case, but scribes have the ability to influence this through clarifying the content of notes. This suggests that skills are differentiated in line with the task differentiation.

The standardization of rounds has also been called for [23, 24]. The mechanism approach also suggest ways in which this may occur. Rounds may be structured so that mechanisms are dealt with in a logical order, helping to ensure the completeness and efficiency of each. Given limited time for each patient, one potential improvement may be to identify synergies between mechanisms, such as across the three areas of agreement, that is: information accumulation, understanding and decision-making. The tensions are also relevant to standardized processes. For example, information generation and collection need to balance, as sought information tends towards confirmation and generated information tends towards exploration. Group reasoning and individual reasoning, and idiographic versus nomothetic knowledge input, also both need to balance. Whilst practitioners work with these tensions every day, examining them with respect to mechanisms provides an opportunity to fine tune this balance.

Improving non-technical skills and standardizing processes could be expected to reduce the number and severity of errors. Another way to reduce errors is through treating ward rounds as a type of program and evaluating them. Through evaluation it is possible to identify practice improvements to enhance the effectiveness of rounds and to suggest ways to better educate medical trainees or to provide professional development to experienced practitioners. The mechanisms can be used to create a program theory, which is the first step in theory-based evaluation [45]. Features of the mechanisms can also be used for guiding evaluation, such as by examining the synergies between mechanisms or by identifying desirable features with respect to the themes and mechanisms.

### Limitations

Context is central to the CR worldview. In this study, context has featured within each mechanism, but contexts are unbounded thus any study must be highly selective in its choices of inclusion. In CR, judgemental rationality underpins context selection but alternative choices are always possible. No study should be taken as a final statement but rather as a step on the path to greater understanding, of which subsequent steps are supplied by future studies. The same point may be made about the selection of outcomes, outputs and the mechanisms themselves.

Another scope limitation involves the focus of this project on practitioner reasoning. Ward round reasoning may be viewed from the perspective of the patient, the hospital administration, other stakeholders such as nurses or allied health practitioners, or various other possibilities. Whilst it is always necessary to restrict the focus for practical purposes, alternative viewpoints may find alternative results of equal validity.

Another limitation concerns the consolidation of mechanisms. The mechanisms presented in this paper are characterized as somewhat atomistic. This reductionism is justified as a technique for identifying discrete mechanisms as a first step, but in reality mechanisms coexist and continually interact. The mechanisms are constructs and, having been identified and found to be sensible, they should be put to practical use, such as to explain reasoning as a whole. This necessarily involves recognizing and theorizing about their interactions.

### Future research and applications

As already noted, contextual factors should be the subject of future research. This may involve exploring the influence of alternative macro contexts, such as hospitals in varying geographical locations or specialized wards. Alternative micro contexts are also of interest, such as varying case types, interpersonal combinations or practitioner profiles. More detailed explorations of the mechanisms in similar contexts is also warranted.

Another direction for future work includes the development of models that consider the mechanisms collectively or as part of a system, to help explain how ward rounds function as an integrated practice, particularly through understanding the dynamic interplay of mechanisms.

As results are always contingent, modelling and theorizing should continue indefinitely and results applied on a case-by-case basis. Despite this caveat, mechanisms are sufficiently regular to allow for modelling, which may then inform practical activities. As already mentioned, these may include ward round evaluation, process improvement, practitioner education and skill development.

## Conclusion

This research aimed to improve the understanding of ward rounds through exploring the program theory of collaborative group reasoning therein. This was represented by mechanisms within three categories; information accumulation, sense-making and decision-making. Information accumulation is internally focused and relies on combining collective and generative modes, sense-making relies on constructing and disrupting understanding, and decision-making involves balancing option generation and selection. Within each of these categories three distinct mechanisms were identified, involving sharing, agreeing and recording information.

Themes that cut across all mechanisms were discussed, concerning time constraints, practitioner hierarchies, tasks associated with professional roles, criteria used in reasoning, and tensions associated with group reasoning. This representation of the program theory of ward rounds provides a basis for understanding reasoning in rounds, from which theory-based evaluation tools may be developed and improvements to practice identified, thus supporting enhanced medical outcomes for patients.

## Abbreviations

CR: Critical realism
NDM: Naturalistic Decision Making
